# Hematopoietic Stem Cell Transplantation for the Treatment of Epstein-Barr Virus-Associated T- or NK-Cell Lymphoproliferative Diseases and Associated Disorders

**DOI:** 10.3389/fped.2018.00334

**Published:** 2018-11-06

**Authors:** Akihisa Sawada, Masami Inoue

**Affiliations:** Department of Hematology/Oncology, Osaka Women's and Children's Hospital, Izumi, Japan

**Keywords:** CAEBV, EBV, HLH, LPD, PTLD, HSCT

## Abstract

Chronic active Epstein-Barr virus infection (CAEBV) is a prototype of EBV-associated T- and/or NK-cell (EBV^+^ T/NK-cell) lymphoproliferative disorders. Most subtypes of these are lethal. We established a unified treatment strategy composed of step 1 (immunochemotherapy: steroids, cyclosporine A, and etoposide), step 2 (multi-drug block chemotherapy), and step 3 (allogeneic hematopoietic stem cell transplantation; HSCT) for CAEBV and its related diseases. Allogeneic HSCT is the only cure for CAEBV with few exceptions. Primary-EBV infection-associated hemophagocytic lymphohistiocytosis (primary-EBV HLH) is also an EBV^+^ T/NK-cell lymphoproliferation. The nature of EBV^+^ T/NK cells in CAEBV and those in primary-EBV HLH differ. In primary-EBV HLH, most patients need step 1 only and some require step 2 for the successful induction of apoptosis in EBV-infected T cells; however, some exceptional patients require HSCT. We herein present our single institutional experience of CAEBV and primary-EBV HLH, together with that of post-transplant EBV^+^ T/NK-cell lymphoproliferative disease. We also discuss some practical points on HCST with a review of the literature.

## Introduction

Epstein-Barr virus (EBV), a B-cell lymphotropic virus, was revealed to have the potential to infect T and NK cells and cause lymphoproliferative diseases (LPDs) in 1988 and 1989 (1–4). Since then, as the prototype of these LPDs, various efforts have been made to diagnose and treat chronic active EBV infection (CAEBV) and investigate its etiology and pathophysiology. The natural clinical course of these LPDs with only supportive care is lethal with some exceptional disease subtypes. Allogeneic hematopoietic stem cell transplantation (HSCT) has been the most reliable radical treatment. However, it has a mortality rate of ~10%, and a severe morbidity rate of another 10%. It is challenging to select an adequate treatment before HSCT, identify which patients need HCT, and establish which type of HSCT is appropriate. We herein present our current strategy, which has been developed by an institutional review, and discussed it with a review of the literature.

The current classification of EBV-associated T- and/or NK-cell (EBV^+^ T/NK-cell) lymphoproliferative disorders (5, 6) and their requirement for HSCT are listed in Table 1. The treatment is the same between CAEBV and its related diseases, i.e., hypersensitivity to mosquito bites (HMB) and severe-type hydroa vacciniforme (sHV). The main targets of our current literature are CAEBV, its related diseases, and primary-EBV infection-associated hemophagocytic lymphohistiocytosis (primary-EBV HLH); however, EBV^+^ T/NK-cell lymphomas or leukemia and post-transplant EBV^+^ T/NK-cell lymphoproliferative disease (T/NK-LPD) are also referred. This retrospective study was approved by the Research Ethics Committee of Osaka Women's and Children's Hospital (the name has been changed: previously, Osaka Medical Center and Research Institute for Maternal and Child Health).

**Table 1:** EBV-associated T/NK-cell lymphoproliferative disorders.

**Category**	**Disease**	**Requirement for HSCT**
1. Acute/transient	Primary-EBV infection-associated hemophago-cytic lymphohistiocytosis (Primary-EBV HLH)	Some
	Classical hydroa vacciniforme (cHV)	None
2. Chronic/progressive	Chronic active EBV infection (CAEBV)	Nearly all
	Hypersensitivity to mosquito bites (HMB) (Mosquito bite allergy; MBA) Severe-type hydroa vacciniforme (sHV)	
3. Malignant	Aggressive NK-cell leukemia (ANKL)	Mostly
	Extranodal NK/T-cell lymphoma, nasal type (ENKTL) Hepatosplenic T-cell lymphoma Peripheral T-cell lymphoma, not otherwise specified (PTCL, NOS)	
4. Others	Post-transplant EBV-associated T/NK-cell lymphoproliferative disease (EBV^+^ T/NK-PTLD)	Uncertain

## Overview of CAEBV

### Background

CAEBV is a chronic, but progressive and lethal disease with persistent/recurrent infectious mononucleosis (IM)-like symptoms (7). Kawa et al. developed a treatment strategy for CAEBV based on their early etiological and pathophysiological findings (3, 4). In their early studies, IL-2 transiently controlled the symptoms of patients with CAEBV; however, this effect was only observed in a limited number of patients (8). Steroid monotherapy, or later with cyclosporine A (CsA), resulted in the remission of symptoms in most patients, and etoposide (Etp) was effective for CAEBV-associated HLH. However, without radical treatment, all patients died of disease progression: uncontrollable HLH flare followed by distributive shock or multi-organ failure, affected-cell infiltration resulting in organ failure, or disease progression to a refractory malignant lymphoma/leukemia (9–11).

Based on the idea that CAEBV is not a simple infection of EBV, but an EBV-associated neoplasm, they administered and developed multi-drug block chemotherapy comprised of anti-cancer drugs, and finally allogeneic HSCT with revolutionary success (12). These ideas and treatment concepts were gradually accepted and are now widely employed, and CAEBV is partially cited in the WHO classification in 2008 and revised classification in 2017.

### CAEBV-related diseases

In HMB and sHV, systemic IM-like symptoms are induced with a topical skin reaction by mosquito bites and sunlight, respectively. HV is a skin disease induced by sunlight, which generally regresses spontaneously within several years (13, 14). However, some patients with HV exhibit disease progression, in which systemic manifestations are also induced by sunlight, and die with a CAEBV-like clinical course. The former is nomenclated as classical HV (cHV), and the latter as sHV.

Patients with HMB manifest a cutaneous ulcer, with a systemic reaction as the disease progresses, induced by a mosquito sting. In contrast to HV, all patients with HMB follow a CAEBV-like lethal clinical course (15–17). HV and HMB were revealed to be EBV^+^ T/NK-LPDs, and sHV and HMB are regarded as CAEBV-related diseases and treated using the same strategy as that for CAEBV.

## Treatment of CAEBV: a single institutional review

### Unified treatment strategy

**Figure 1:**
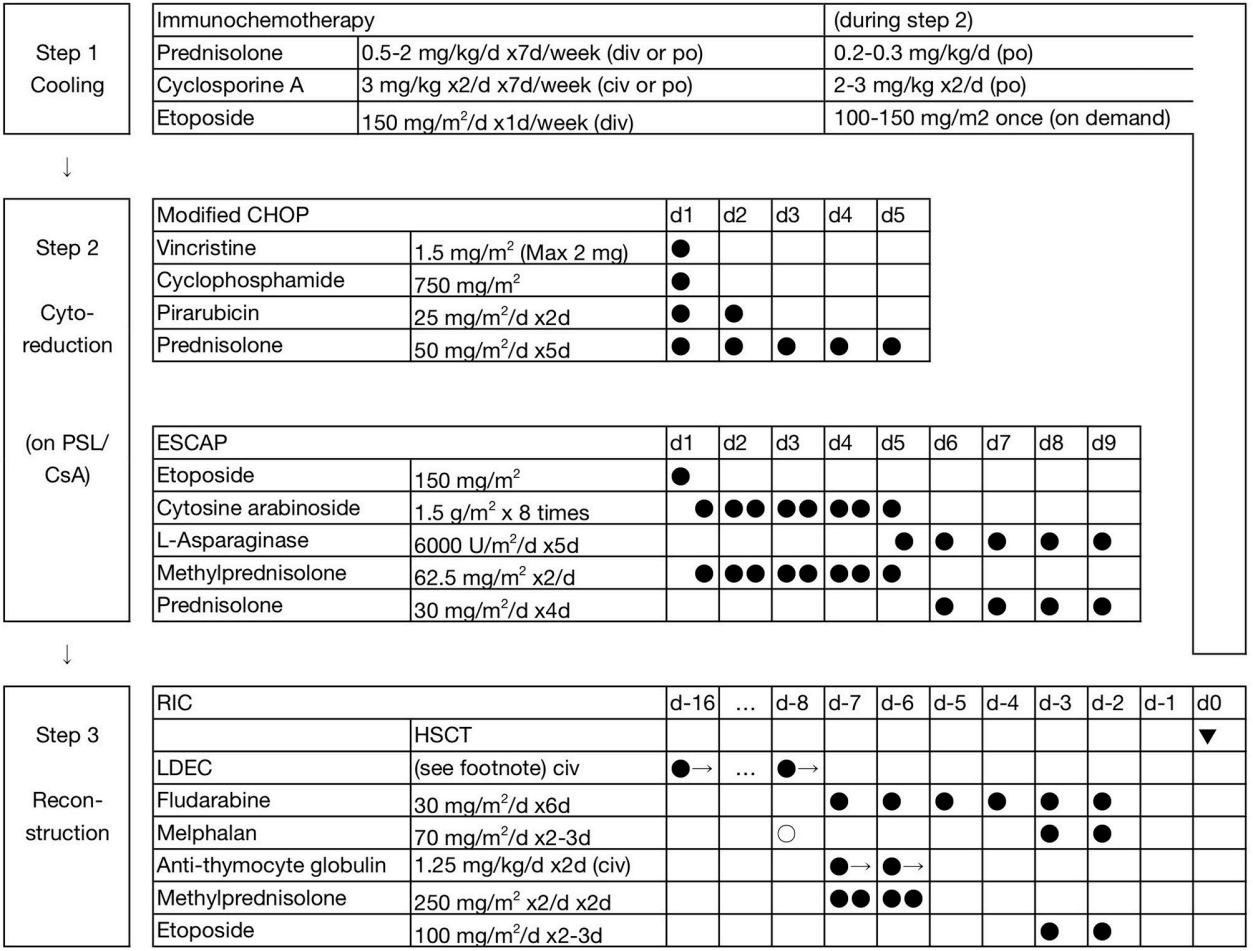
Three-step strategy for the treatment of EBV + T/NK-LPDs. In step 1 (cooling), the initial dosage of prednisolone (PSL) is 1–2 mg/kg/d for children and 0.5–1 mg/kg/d for adults. Etoposide (Etp; 150 mg/m^2^ or 5 mg/kg, weekly) is omitted in patients without symptoms of HLH. In step 2 (multi-drug chemotherapy), lower doses of PSL and cyclosporine A (CsA) were maintained, particularly for patients with a higher burden of residual disease. LDEC: low-dose Etp 30 mg/m^2^/d and cytosine arabinoside 20 mg/m^2^/d are continuously administered for 24 h for 1.5 (0.5–2) weeks before the initiation of RIC. Closed circles indicate fixed administration, and open circles indicate optional administration. One precedent dose of melphalan 70 mg/m^2^/d is added (total 210 mg/m^2^/d) for children and adolescents at a high risk of rejection, and is replaced by systemic irradiation of 3 Gy with gonadal blockade in adults.

The unified treatment strategy for CAEBV (with and without HLH), sHV, and HMB is shown in Figure 1 [F]. The initial treatment is immunochemotherapy (step 1). At the onset of HLH, it is sometimes difficult to make a differential diagnosis between primary-EBV HLH and HLH as a manifestation of CAEBV, other types of EBV^+^ T/NK-LPDs, or even severe IM (a kind of acute/transient EBV^+^ B-LPDs). Etp was omitted for patients without symptoms of HLH. In CAEBV and its related diseases, for a radical treatment, most patients are moved to multi-drug block chemotherapy (step 2) within 1–2 weeks.

Patients received 2–3 courses of chemotherapy on average in step 2. During the interval of chemotherapy, lower doses of prednisolone (PSL) and CsA were maintained, particularly in patients with a higher burden of residual disease. First-line chemotherapy is modified CHOP (Figure 1). Second-line chemotherapy is ESCAP. However, patients with complications may undergo the Capizzi regimen instead: high-dose cytosine arabinoside (HDCA) 3 g/m^2^ 4 times (every 12 h), L-asparaginase (L-Asp) 10,000 units/m^2^ once (4 h post-CA), and PSL 30 mg/m^2^/d (days 1 and 2). The interval is typically 3–4 months between the initiation of treatment and allogeneic HSCT (step 3).

### Significance of multi-drug chemotherapy

We published our single center experience of 77 patients with CAEBV and its related diseases, HMB and sHV, along the 3-step strategy (6). The EBV-DNA load in whole peripheral blood (PB) is a practical and useful marker for residual disease in CAEBV and its related diseases (HMB and sHV). Effective chemotherapy is defined as a reduction of ≤ 1/10, measured with quantitative PCR, by each course of chemotherapy. In 6 out of 77 patients, the EBV-DNA load was reduced to the minimum detection limit (200 copies/mL) or less by several courses of chemotherapy. Two patients underwent successful HSCT under their parents' and physician's choice, another 2 patients exhibited early elevations in the EBV load and successfully underwent HSCT, and the remaining 2 patients (CAEBV 1 and HMB 1) received one additional course of effective chemotherapy and have been in continuous complete remission (CR) without HSCT (6, 18).

The necessity for and significance of multi-drug chemotherapy (step 2) is under debate. In our strategy, multi-drug chemotherapy is important as a preparation for subsequent allogeneic HSCT. It provides (1) a safe bridge to HSCT by suppressing disease activity, (2) a high rate of engraftment of donor cells at HSCT, and (3) a low relapse rate after HSCT (6). The majority of patients with CAEBV died of disease within observation periods unless they received radical treatment. Some physicians consider there to be exceptional cases without disease progression and organ failure. We hypothesize that exceptional cases may be present in the subgroup of patients achieving complete molecular CR by multi-drug chemotherapy.

### Allogeneic HSCT

In our institute, 75 patients had the intention to receive HSCT. Of these, 12 patients progressed to an emergency medical condition. Therefore, 63 patients who underwent planned transplantation state were involved in the following analysis. Reduced-intensity conditioning (RIC) was superior to myeloablative conditioning (MAC) (19), and revised 3-year overall survival rates (3y-OS) were reported to be 90.7 ± 4.0% (*n* = 54) and 66.7 ± 15.7% (*n* = 9), respectively (*p* < 0.05) (6). Furthermore, the incidence of late sequelae after RIC, such as gonadal dysfunction, may be lower than that after MAC (20). The current RIC regimen (standard RIC) includes a total melphalan (LPAM) dose of 140 mg/m^2^, as shown in Figure 1.

Cord blood transplantation (CBT) and bone marrow transplantation (BMT) are excellent sources of HSCT, and 3y-OS were 93.3 ± 6.4 and 92.9 ± 6.9%, respectively (*p* = 0.87); however, the incidence of engraftment failure was higher in CBT (21). RIC for CBT was successfully augmented thereafter, and no engraftment failure has since been observed (*n* >10) (6). The current augmentation of RIC for CBT is LPAM 70 mg/m^2^ on day-8 in children and adolescents, and systemic irradiation with 3 Gy with gonadal blockade in adults if a recipient has received only 2 or 3 courses of chemotherapy before CBT.

## HSCT for CAEBV in various situations

### Adult-onset CAEBV

CAEBV is now recognized to occur not only in children and adolescents, but also in adults at any age. Half of the children (including adolescents) with CAEBV died in 5 years, and most of them died in 10–15 years without radical treatment (10). Two studies reported that adult-onset CAEBV progresses rapidly, and most of patients died within 5 years (22, 23). In our series, 3y-OS was equivalent between adults (≥ 20 years of age at onset) and children (71.4 ± 12.1, 76.6 ± 5.3%, respectively; *p* = 0.61) (6). Therefore, we concluded that our 3-step strategy is also applicable to adults. Arai et al. reported similar findings (OS 61.5%) for adult-onset CAEBV (24).

### Emergent HSCT

In our series, in contrast to the promising findings of planned HSCT (*n* = 63; 3y-OS 87.3 ± 4.2%), most patients with advanced/uncontrollable disease (*n* = 12), including 8 who managed to undergo emergent HSCT, were not rescued (3y-OS 16.7 ± 10.8%) (6). Our findings were consistent with those by Arai et al. who reported that OS was 100% after HSCT for inactive disease, but was 0% after HSCT for active disease (24).

Patients with liver-transaminase elevations or hyperferritinemia were restored by HSCT, i.e., remedial HSCT (2 out of 5 survived). However, HSCT did not save patients (compassionate HSCT, 0 out of 3 survived) with severe jaundice (liver failure), anuria (renal failure), or tracheal intubation (due to distributive shock after HLH flare); these difficult cases were attributed to disease progression and not to chemotherapy or age (6). Therefore, we consider that initiating treatment earlier to complete HSCT in advance leads to higher survival, although HLH flare or disease progression may occur at any time, even under treatment.

## Primary-EBV HLH

### Background

Primary-EBV HLH is a secondary HLH following a primary EBV infection; secondary means that it occurs in children (and occasionally in adolescents and young adults) without known immunodeficiencies, including familial HLH (FHL). It has a lethal potential for HLH flare followed by multi-organ failure without an adequate treatment. These more severe manifestations of primary-EBV HLH than those of other infection-induced HLH may be attributed to primary-EBV HLH not being simple infection-induced HLH, but LPD-associated HLH based on EBV^+^ T/NK-cell proliferation (typically CD8^+^ T cells). The EBV infection of T cells in primary-EBV HLH has also been reported in non-Asian children (25).

The majority of patients with primary-EBV HLH are simply cured with immunochemotherapy (steroids, CsA, and Etp), such as the FHL-oriented protocol (HLH-94 or HLH-2004) or our step 1 (26, 27). Remission was achieved by immunochemotherapy in 86–90%, and recurrence was observed in 8–17% (28, 29). Notably, eight out of the 9 patients (89%) who did not achieve remission during the initial steroid treatment/CsA/Etp died (29). Therefore, allogeneic HSCT is required for patients refractory to immunochemotherapy. In prospective studies including a small ratio of patients with a congenital gene mutation responsible for HLH (2–8%), HSCT was administered to 15–23% of patients (27, 28). OS was 76–90% (28, 30), and varied between 53 and 86% in patients who underwent HSCT (27, 28, 31, 32).

In prospective studies, patients with severe/persistent or recurrent disease moved to HSCT without multi-drug chemotherapy (33). In contrast, in practical situations, ~50% of patients were treated with multi-drug chemotherapy before HSCT (32). However, few studies have been published from the viewpoint of the effectiveness of multi-drug chemotherapy. In one study on 20 patients, 13 achieved remission after steroids with or without CsA and/or Etp (34). Thereafter, 4 out of the remaining 7 patients achieved remission by multi-drug chemotherapy (mostly CHOP) and continued to be disease-free without HSCT.

Prolonged high doses of steroids/CsA/Etp may lead to a severe immunodeficiency, and result in the occurrence of another complication, immunodeficiency-associated B-LPD (35). If immunodeficiency-associated B-LPD can be diagnosed early, rituximab may be effective. A systematic review and meta-analysis showed that survival ratios were 68 and 80% in the immunochemotherapy (without HSCT) group and HSCT group, respectively, and concluded that both approaches equally contributed to decreasing mortality (36); however, disease severity may differ in each group.

### A single institutional review

We treated 23 patients with primary-EBV HLH between 2000 and 2017 since the measurement of the EBV-DNA load with quantitative PCR became available (paper under preparation). In our institutional review, a bias was expected to exist in disease severity; our present study may include a higher ratio of severe patients than others because, as an example, 50% of severely affected patients were referred from areas outside our prefecture.

Patients with primary-EBV HLH follow the unified treatment strategy shown in the section on CAEBV (Figure 1). Most patients with severe HLH required pulsed high-dose methylprednisolone (HD-mPSL; 0.5 g/m^2^/d for 3 days) and Etp (150 mg/m^2^/d or 5 mg/kg/d one day per week). In contrast, the delayed initiation of and/or dose reductions in CsA were allowed for the patient conditions. Patients start the tapering of PSL within 1–2 weeks if they achieve the remission of symptoms, whereas those refractory to step 1 move on to step 2.

Treatment responses varied; 9 patients needed only step 1 (group A), 6 needed steps 1 and 2, but did not require HSCT (group B), and the remaining 8 needed steps 1, 2, and 3 (group C). OS also varied; all patients (15/15) are alive and well in groups A and B, in contrast to only 3/8 (38%) in group C. In group A, 2 patients showed a complete response to PSL and CsA without Etp, and 7 received Etp (mainly once or twice). Patients refractory to or dependent on immunochemotherapy (step 1) were administered multi-drug chemotherapy (in groups B and C). First-line chemotherapy was modified CHOP, and second-line chemotherapy contained HDCA and/or L-Asp (Figure 1).

Together with groups B and C, 6 out of 14 patients successfully avoided allogeneic HSCT. Patients with resistant disease against multi-drug chemotherapy were regarded as absolute candidates for allogeneic HSCT. One severe patient omitted multi-drug chemotherapy for urgent HSCT. Among the 8 patients in group C, 5 had early deaths (mainly due to HLH), 1 relapsed 12 months after RIC followed by BMT (RIC-BMT), but was successfully rescued by MAC-CBT, and 2 maintained CR after HSCT.

### Absolute eligibility for HSCT in primary-EBV HLH

In our series, group B included 3 patients who were referred to our institute requiring further treatment than steroids/CsA/Etp, i.e., allogeneic HSCT, but successfully substituted multi-drug chemotherapy for HSCT. As a corollary, patients in group C were absolutely eligible for HSCT. Similar to CAEBV, one of the roles of multi-drug chemotherapy is to identify who definitely needs HSCT and who does not among patients with primary-EBV HLH.

Group C may form a distinct disease subset. In some patients, disease activity progressed after a significant reduction in the EBV load or even after achieving complete donor chimerism by HSCT. These patients in group C had no mutation in the known genes responsible for FHL or other primary HLH. They possibly had other unknown gene mutations in T/NK cells and/or non-T/NK cells that predisposed them to the induction/maintenance of progressive HLH (37–39).

## Other types of EBV^+^ T/NK-LPDs

### Post-transplant EBV^+^ T/NK-LPD

Typical post-transplant LPD (PTLD) has been described as EBV-associated and B-cell type LPD, occurring before 12 months (particularly in the third month) after HSCT or at any time after organ transplantation (40, 41). Typical PTLD is susceptible to rituximab. In contrast, T/NK-PTLD is a dismal complication after transplantation (OS was ~20%) (42). However, EBV^+^ T/NK-PTLD had a better prognosis than EBV^−^ T/NK-PTLD (OS of ~38 and 13%, respectively) (42).

As one atypical PTLD, we have had 3 recipients with late-onset EBV^+^ T/NK-PTLD including one patient reported previously (patient 1) (18). All 3 patients responded to HDCA- and/or L-Asp-containing chemotherapy, and are in continuous CR without HSCT (paper under preparation). Although this atypical PTLD belongs to the variety of EBV^+^ T/NK-LPDs, a patient subset may exist that responds to chemotherapy and may be cured without HSCT.

### Malignant EBV^+^ T/NK-LPDs

Our patient number was limited in this subset. In the literature, more than half of patients with localized extranodal NK/T-cell lymphoma of the nasal type (ENKTL) were successfully treated with local irradiation and chemotherapy (2y-OS 78%) (43). Malignant T/NK-cell lymphoma/leukemia is susceptible to HDCA and/or L-Asp, such as the SMILE regimen (44–47). However, the SMILE regimen was highly toxic in elderly patients or those with organ dysfunctions (48). Patients with advanced ENKTL or aggressive NK-cell leukemia (ANKL) are treated with the SMILE regimen and HSCT (46, 47); however, OS is still dismal, particularly in ANKL (1y-OS <30%).

## Difference between primary-EBV HLH and CAEBV

### Proapoptotic potential in EBV-infected cells of primary-EBV HLH

In primary-EBV HLH, treatment is tapered alongside the remission of symptoms. Residual disease of primary-EBV HLH, molecularly assessed by the EBV-DNA load with quantitative PCR, may be significantly reduced with successful treatment; however, molecular CR (EBV < 200 copies/mL in PB) and the complete clearance of EBV-infected T/NK cells (typically CD8^+^ T cells) were not mandatory at the end of the treatment, and minimal residual disease (MRD) disappeared within a half or a couple of years. This was in contrast to CAEBV, for which a positive result for MRD without HSCT resulted in overt relapse.

Investigators failed to infect peripheral blood T/NK cells from healthy volunteers (PB-T/NK cells) with EBV *in vitro*, except in one study (49). Furthermore, although EBV managed to infect NK-cell lines and PB-T/NK cells, most were lost due to apoptosis within 72 h (49). In two studies using clinical specimens, EBV-infected T cells and NK cells were detected in 0–50 and 50–100% of patients with IM, i.e., initial EBV infection, respectively (50, 51), but are rarely maintained and merely result in T/NK-LPD. These findings are consistent with our previous observations, and provided a rationale for our recent treatment.

### Different nature between primary-EBV HLH and CAEBV

**Figure 2:**
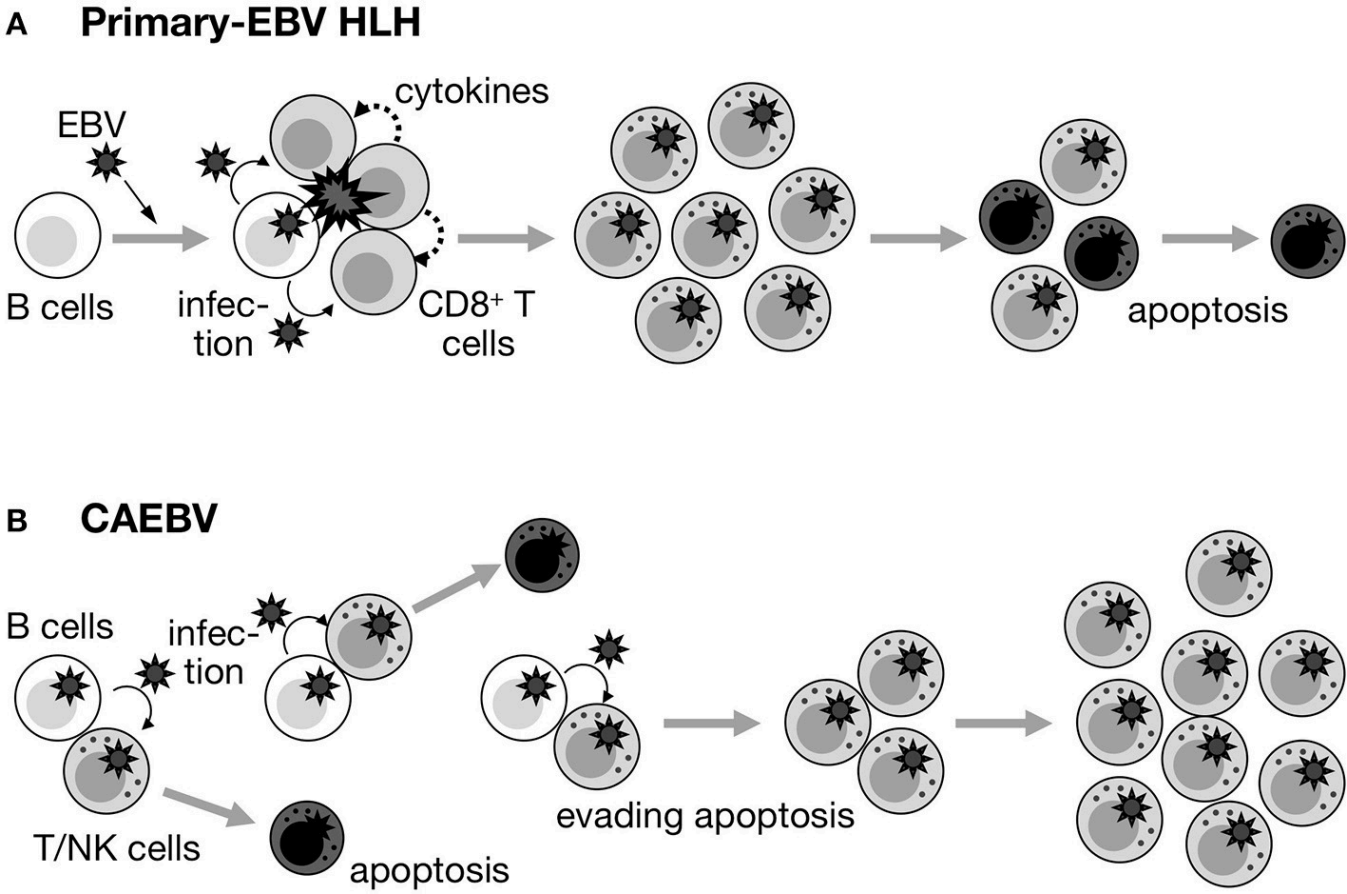
Fate of EBV^+^ T/NK cells in primary-EBV HLH and CAEBV. **(A)** Primary-EBV HLH. EBV-infected T/NK cells may transiently proliferate under specific conditions (primary EBV infection and large amounts of cytokines). However, they maintain a proapoptotic nature, and may be induced to enter apoptosis by themselves, steroids/CsA, and/or anti-cancer drugs. **(B)** CAEBV. Although EBV occasionally infects T/NK cells, EBV-infected T/NK cells inherently have pro-apoptotic effects and repeatedly appear and disappear. However, some of these cells acquire a self-maintaining and self-expanding predisposition over the course of years and contribute to the development of disease.

Our observations and the findings described above prompted us to speculate that the nature of EBV-infected T/NK cells in primary-EBV HLH and CAEBV completely differs (Figure 2). In primary-EBV HLH, EBV-infected T/NK cells are activated due to primary EBV infection, and maintain an antiapoptotic predisposition due to a specific internal environment, such as a high concentration of cytokines during early EBV infection. Therefore, the purpose of treatment is to induce apoptosis in affected and activated cells, as well as to suppress the T-cell cytokine storm and monocyte/macrophage hyperinflammation. In contrast, EBV-infected T/NK cells in CAEBV have acquired a self-expanding nature during years of evading eradication (Figure 2), and the purpose of treatment is complete cell death (total cell killing).

### Spectrum between CAEBV and malignant T/NK-LPDs

CAEBV shares some common features with ANKL and ENKTL, revealing a spectrum between CAEBV and malignant EBV-associated T/NK-LPDs (52, 53). Difficulties are associated with making a differential “subdiagnosis” between CAEBV and malignant EBV^+^ T/NK lymphoma/leukemia (ANKL and ENKTL) in some cases. However, their distinction may be practically less important and the diagnosis of “EBV-associated T/NK-LPD” may be sufficient for a severely ill emergent patient because both need similar therapeutic approaches that include allogeneic HSCT (5). In contrast, EBV^+^ T/NK cells in PTLD, arising under some immunodeficient conditions, particularly after transplantation, may reserve the potential for apoptosis and susceptibility to chemotherapy.

## Other considerations for HSCT

### Alloimmunity as the main effector against CAEBV after HSCT

The relapse rate after planned HSCT among patients with CAEBV was as low as < 5%, although most of their conditioning regimens were RIC rather than MAC (6). The relapse rate did not increase even after the transplantation of CB, which is naïve to EBV (21). In one study, cytotoxic lymphocytes (CTLs) against EBV were induced after allogeneic HSCT in two patients with CAEBV (54). However, CTLs against EBV were not properly induced and EBV DNAemia was often observed after HSCT (21), which may correlate with progression to EBV^+^ B-PTLD, but not with the relapse of CAEBV. In contrast, mixed chimerism or autologous recovery correlated with the relapse of CAEBV (6). These findings prompted us to speculate that alloimmunity is the main effector against EBV^+^ T/NK cells after HSCT, and that the success of allogeneic HSCT over CAEBV mostly depends on allo-reactive CTLs against recipient cells (21).

This is not the case for malignant EBV^+^ T/NK-LPDs. The incidence of recurrence is high, even after allogeneic HSCT, particularly in patients with ANKL. CTL against EBV may have some benefit for these patients as maintenance therapy after HSCT (55).

### Effects of anti-thymocyte globulin on EBV^+^ T/NK-LPD

The purpose of our early administration of low-dose rabbit anti-thymocyte globulin (ATG; Thymoglobulin®, Sanofi, France; 1.25 mg/kg/d on d-7 and d-6) is to reduce recipient T-cell immunity and enforce donor-cell engraftment, and, in addition, reduce EBV-infected T/NK-cell numbers for better disease control.

Sanacore et al. suggested that the early administration of ATG at 4.5 mg/kg 2 weeks before HSCT resulted in a sub-therapeutic ATG concentration (< 1 μg/mL in serum) on day 0 of HSCT; therefore, it only depleted host T cells selectively and enhanced donor-cell engraftment (56). Their pharmacokinetics suggest that the concentration of ATG may be lowered to a sub-therapeutic level on the day of HSCT (day 0), even if ATG is administered at 2.5 mg/kg 1 week before HSCT (i.e., early dosing regimen). Penack et al. reported that although Lymphoglobulin® (equine anti-thymocyte globulin) was less effective for NK-cell depletion, ATG induced apoptosis and necrosis in NK and T cells (57). However, the effects of ATG on NK cells currently remain unknown in clinical settings.

**Figure 3:**
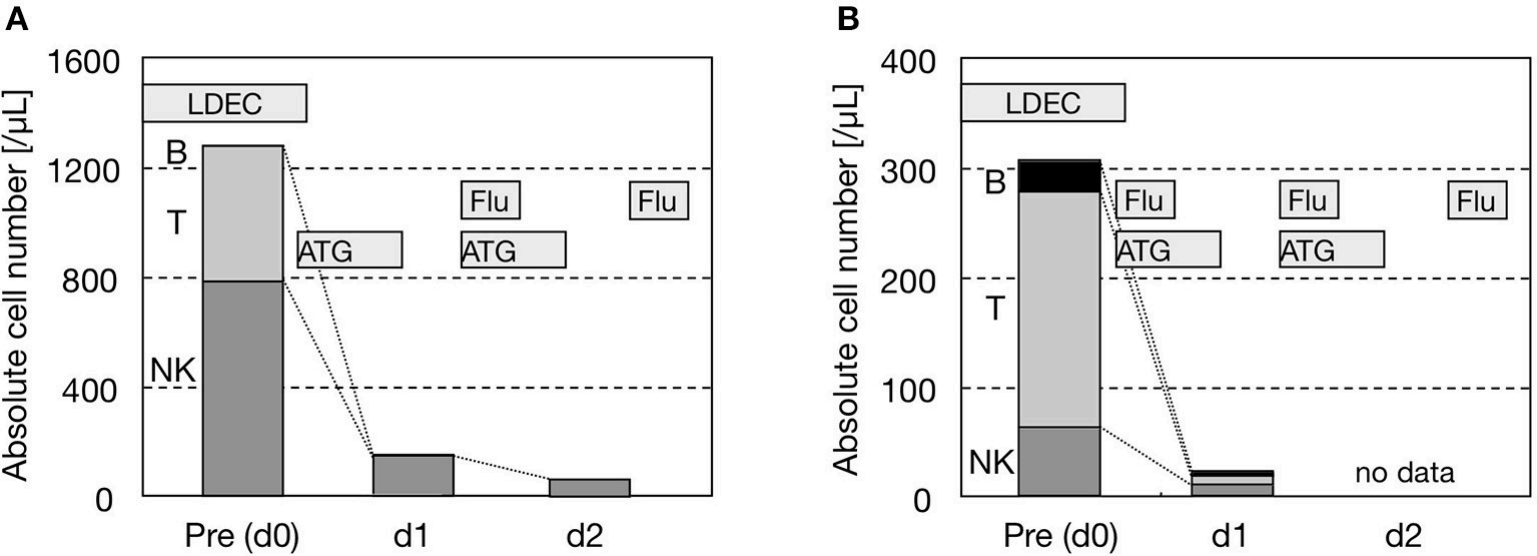
Reductions in NK cells by ATG. **(A)** UPN605. **(B)** UPN612.Both were diagnosed as HMB of the NK-cell type. The absolute number of each lymphocyte subset in PB was counted on days 0, 1, and 2 before and after the administration of ATG as the initiation of RIC. Other drugs were also shown. The lymphocyte subset was analyzed by three-color flow cytometry (FCM) using fluorescein isothiocyanate-, phycoerythrin/rhodamine-, and phycoerythrin-cyanine 5-conjugated antibodies (Beckman Coulter Inc., USA). FCM was performed with EPICS XL® (Coulter Inc., USA). B, T, and NK cells were defined as CD20 + CD19 + CD45 + cells, CD3 + CD45 + cells, and CD3 - CD56 + CD45 + cells, respectively. LDEC, low-dose etoposide and cytosine arabinoside; Flu, fludarabine.

We measured the absolute number of each lymphocyte subset in PB during RIC (Figure 3). In addition to the absolute number of T cells, that of NK cells was also diminished by ATG: <1/5 in 1 day and <1/10 in 2 days after the initiation of ATG. Therefore, although NK cells were counted as a whole without the distinction of EBV-infected and EBV-uninfected NK cells, we regarded ATG to be effective for the disease control of EBV^+^ NK-LPD as well as EBV^+^ T-LPD. As a result, if ATG is administered to patients with a high burden of residual disease, conditioning-induced (possibly ATG-induced) HLH needs to be carefully considered (21). ATG actually induces apoptosis and necrosis in EBV^+^ T/NK cells in clinical settings. However, the cytoreductive effects of ATG on EBV^+^ T/NK cells may be transient. When the complete engraftment of donor cells fails (mixed chimerism or autologous recovery), the EBV load may increase again with hematopoietic recovery after HSCT (6).

## Future directions

Despite the evolution of treatment strategies against EBV^+^ T/NK-LPDs, the prognosis of patients with progressive/refractory disease remains poor. Better methods in step 2 are desired. JAK 1/2 inhibitors may exert prophylactic and therapeutic effects on HLH (58). The SMILE regimen has been used in some hospitals. The novel combination of romidepsin and pralatrexate was found to be effective for T-cell lymphoma (59). Furthermore, bortezomib and ganciclovir have been suggested to reduce the disease burden (60). Cell therapy, such as CTLs against EBV (as acquired immunity) or HLA-haplotype incompatible lymphocytes (for alloimmunity), may also be effective to some extent (55, 61, 62). We consider better disease control with these modalities before and after HSCT to contribute to more promising prognoses.

## Author contributions

All authors listed have made a substantial, direct and intellectual contribution to the work, and approved it for publication.

### Conflict of interest statement

The authors declare that the research was conducted in the absence of any commercial or financial relationships that could be construed as a potential conflict of interest.
